# Padres Preparados, Jóvenes Saludables: A Randomized Controlled Trial to Test Effects of a Community-Based Intervention on Latino Father’s Parenting Practices

**DOI:** 10.3390/nu14234967

**Published:** 2022-11-23

**Authors:** Aysegul Baltaci, Ghaffar Ali Hurtado Choque, Cynthia Davey, Alejandro Reyes Peralta, Silvia Alvarez de Davila, Youjie Zhang, Abby Gold, Nicole Larson, Marla Reicks

**Affiliations:** 1 School of Public Health, University of Minnesota, Minneapolis, MN 55455, USA; 2 School of Public Health, University of Maryland, College Park, MD 20742, USA; 3 Clinical and Translational Science Institute, University of Minnesota, Minneapolis, MN 55455, USA; 4 Center for Family Development, University of Minnesota Extension, Saint Paul, MN 55108, USA; 5 Department of Child and Adolescent Health and Social Medicine, School of Public Health, Medical College of Soochow University, Suzhou 215123, China; 6 Department of Food Science and Nutrition, University of Minnesota, Saint Paul, MN 55108, USA

**Keywords:** randomized controlled trial, community-based intervention, Latino fathers, father’s parenting practices, lifestyle behaviors

## Abstract

Parenting practices have been associated with adolescent lifestyle behaviors and weight status. Evidence is limited regarding the efficacy of interventions to address father influences on adolescent lifestyle behaviors through availability and modeling practices. Therefore, the purpose of this study was to evaluate changes in father parenting practices after Latino families with adolescents participated in the Padres Preparados Jóvenes Saludables (Padres) program. Time-1 (baseline) and Time-2 (post-intervention) data were used from Latino father/adolescent (10–14 years) dyads enrolled in the Padres two-arm (intervention vs. delayed-treatment control group) randomized controlled trial in four community locations. The program had eight weekly, 2.5-h experiential learning sessions on food preparation, parenting practices, nutrition, and physical activity. Two types of parenting practices (role modeling and home food availability) were assessed by father report via questionnaire for each of 7 lifestyle behaviors, for a total of 14 parenting practices. Linear regression mixed models were used to evaluate the intervention effects. A total of 94 father/adolescent dyads completed both Time-1 and Time-2 evaluations. Significant positive intervention effects were found for frequencies of fruit modeling (*p* = 0.002) and screen time modeling (*p* = 0.039). Non-significant results were found for the other 12 father parenting practices.

## 1. Introduction

Many adolescents in the U.S., including Mexican American and other Hispanic adolescents, have poor dietary behaviors [1,2,3], low levels of physical activity, and frequent screen time [4,5]. These behaviors have been identified as critical behavioral determinants of obesity among adolescents [6]. Childhood obesity increases the risk of developing a variety of health complications and chronic diseases, such as becoming overweight or obese as an adult and developing diabetes, metabolic disorders, and heart disease [7,8]. The current Dietary Guidelines for Americans (DGAs) [9] recommend that U.S adults and adolescents increase their intakes of nutrient-dense foods and a variety of fruits and vegetables while limiting energy-dense foods and beverages to meet the recommended food group and nutrient needs to achieve healthy dietary patterns.

Physical activity is defined as any kind of body movement produced by the skeletal muscles that substantially increases energy expenditure [10]. Adequate physical activity during adolescence may contribute to various short- and long-term benefits for the health and wellbeing of adolescents, including a higher level of cardiorespiratory fitness, stronger muscles and bones, lower body fat, and lower symptoms of depression compared to having an inactive lifestyle [11,12]. On the other hand, screen time is a common sedentary behavior among adolescents in the U.S. [13]. Screen time can contribute to increased risk of adiposity, elevated serum triglyceride concentrations, and metabolic syndrome in adolescents [14].

Parenting practices, which influence these health behaviors, have been defined as intentional or unintentional behaviors/actions by parents that shape their child’s attitudes, behaviors, or beliefs [15]. Previous studies have highlighted the positive influence of parenting practices on adolescents’ dietary behaviors [16], physical activity, and screen time [17,18,19]. Several qualitative and cross-sectional studies have shown that Latino parents play a positive role in improving older children and adolescents’ lifestyle behaviors [20,21,22,23,24,25,26] and weight status [27,28] by engaging in positive food parenting practices. However, these studies have primarily focused on Latino mothers and their adolescents, and little research is available assessing father influences on adolescent lifestyle behaviors and health outcomes.

The limited literature available on the influence of Latino father parenting practices on adolescent behaviors has shown positive findings [20,25,28]. A cross-sectional study with 81 Mexican-origin fathers and children aged 7–13 years who participated in the Entre Familia: Reflejos de Salud study showed that children consumed more fruit and vegetables when their fathers used feeding-related reinforcement of healthy eating more frequently [25]. Another study with 174 mother-father-child triads (8–10 years of age) demonstrated that a father’s healthy BMI was related to a child’s healthy BMI z-score [28]. Latino fathers of adolescents reported in focus groups that role modeling and making healthy food and physical activity opportunities available were parenting practices that could help adolescents have healthier food and activity behaviors [21]. Therefore, interventions that focus on Latino father parenting practices to promote healthy lifestyles among Latino youth may be beneficial.

The primary goal of the Padres Preparados, Jóvenes Saludables (Padres) (Prepared Parents, Healthy Youth) program was to prevent overweight and obesity among Latino adolescents in low-income households by increasing the frequency of healthy father food and activity parenting practices [29]. The Padres program was adapted from a successful community-based parenting skills education program to prevent substance use among Latino parents and adolescents [30]. The program was grounded in social cognitive theory [31,32] and based on the principles of community-based participatory research [33], with collaboration from community partners in all design and implementation processes [20]. The aims of the current study were 1) to determine baseline (Time-1) to post-intervention (Time-2) changes in father parenting practices (role modeling and making foods and physical activity opportunities available) in a RCT study with intervention and delayed-treatment control groups, and 2) to assess the intervention effect on changes in father parenting practices from Time-1 to Time-2, adjusted for father age and adolescent age and sex.

## 2. Materials and Methods

### 2.1. Study Design and Sample

This study used Time-1 (baseline) and Time-2 data (after the 8-week program was conducted for the intervention group) from the Padres program trial [29]. The primary outcomes of the randomized controlled intervention trial (identifier: NCT03641521), which were father and adolescent dietary intake and weight status, are reported elsewhere, as well as intervention details [29]. This paper reports on secondary outcomes regarding the frequency of father parenting practices.

Latino fathers or male caregivers (hereafter referred to as fathers) of adolescents 10–14 years, who identified as Latino, spoke Spanish, and had meals at least three times a week with their adolescents were eligible for the study. Families were recruited using social media, flyers, and announcements at community service centers and churches primarily serving low-income Latino families. Fathers and adolescents completed surveys in person at Time-1 and Time-2. The study protocol was approved by the University of Minnesota Institutional Review Board (project identification code: 1511S80707).

### 2.2. Intervention

Father/adolescent dyads were randomized to either an intervention or a delayed-treatment control group [29]. The program was implemented in person at four locations (community service centers and churches) in the Minneapolis/St. Paul metropolitan area between September 2017 and December 2019. In-person implementation was discontinued in March 2020 and not resumed because of public health efforts to limit the transmission of COVID-19. During the intervention, fathers and adolescents attended eight weekly, 2.5-h experiential learning sessions facilitated by bilingual Latino educators. In each session, fathers and adolescents participated together in activities to prepare food, be physically active and learn about nutrition and active lifestyles. In separate parts of each session, fathers participated in activities to develop parenting skills and improve frequency of parenting practices, while adolescents participated in activities to reinforce learning about healthy lifestyle behaviors.

### 2.3. Participation in Evaluation Data Collection

**Figure 1:**
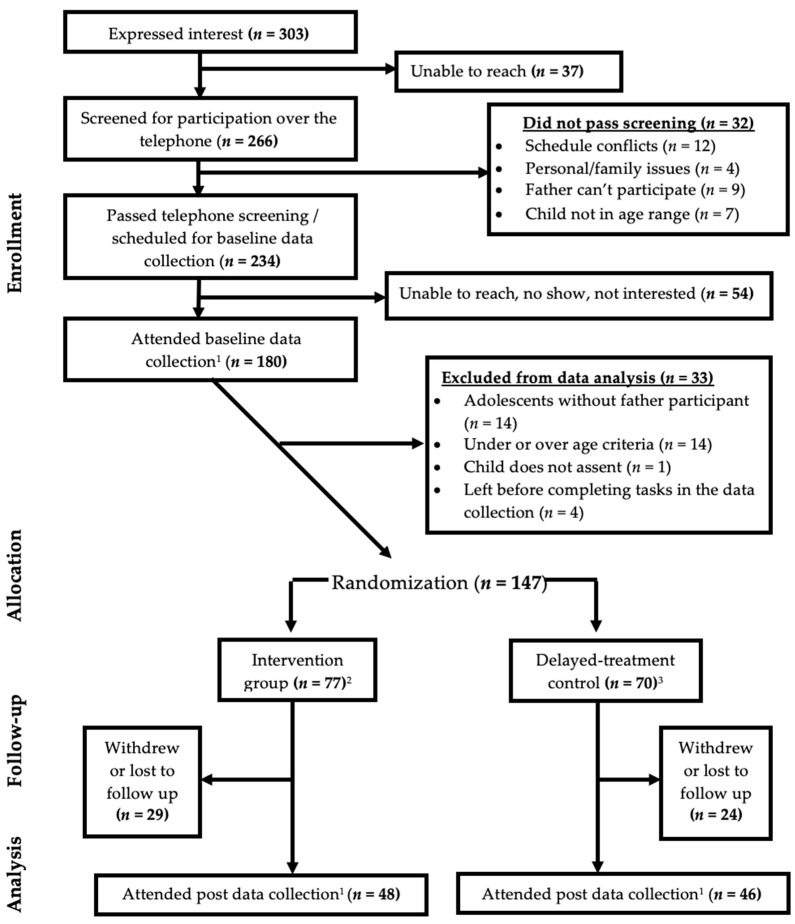
CONSORT diagram (Father/adolescent dyads) [29]. ^1^ Participants reported frequency of paternal parenting practices; ^2^ Four participant dyads were randomized to intervention but attended delayed-treatment control group educational sessions; ^3^ Seven participant dyads were randomized to delayed-treatment control but attended intervention group sessions.

A total of 303 father-adolescent dyads expressed interest in participating in the study (Figure 1). Of those, 266 were screened for eligibility over the telephone and 234 were identified as eligible. Of the 234 dyads, 54 did not attend the Time-1 data collection session, and 20 dyads did not complete the data collection procedures. Time-1 data collection sessions were completed by 147 father/adolescent dyads, with 94 father/adolescent dyads completing both Time-1 and Time-2 data collection.

### 2.4. Sociodemographic Characteristics

Fathers reported their age, years in the U.S., education, employment status, marital status, family annual income, and language spoken at home via surveys. For ease in describing characteristics, education was categorized as middle school or lower, GED (equivalent to a high school diploma) or high school, and some college or higher. Employment was collapsed into four categories: self-employed, unemployed, employed part-time, and employed full-time. Marital status was categorized as single or married/living with a partner.

Adolescents reported their own birthdates and sex. Adolescent age at Time-1 was calculated by subtracting the birthdate from the date of Time-1 data collection divided by the number of days each year (365; 366 for leap years).

### 2.5. Anthropometric Measurements

Adolescents’ and fathers’ body weight and height were measured separately twice in a private space using a digital scale (BWB-800 Scale, Tanita) and a stadiometer by a trained research assistant, according to standardized procedures of the National Health and Nutrition Examination Survey (NHANES) [34]. Two measures of both weight and height were averaged to obtain mean weight and height. Fathers’ body mass index (BMI) was calculated using weight (kg) divided by height squared (m^2^). Adolescents’ BMI percentiles were generated by a SAS program using the 2000 CDC Growth Charts [35].

### 2.6. Father Food and Activity Parenting Practices

The frequency of two types of parenting practices (role modeling and home availability of food or activity opportunities) was assessed by father report via questionnaire for each of seven lifestyle behaviors (fruit, vegetable, sugar-sweetened beverage (SSB), fast food, sweets/salty snack consumption, and frequency of physical activity and screen time) for a total of 14 father food and activity parenting practices. The questionnaire had a total of 33 items, including two items for each of seven role modeling scales, three items for each of six food/activity availability at home scales, and one item for screen time availability at home. Parenting practice questions were developed based on findings from focus groups with Latino fathers [20] and existing validated scales [36,37,38]. Father food and activity parenting practice items and scales showed adequate criterion validity in a preliminary study and internal consistency for all scales based on Cronbach’s α coefficients >0.7 [39].

Fathers were asked two questions about role-modeling frequency, separately, for fruit, vegetable, SSB, fast food, and sweets/salty snack consumption and physical activity and screen time, including (1) how many times fathers were seen by adolescents consuming each type of food or beverage or engaging in physical activity, and (2) how many times fathers consumed each type of food or beverage and engaged in physical activity with adolescents. Response options were almost never or never = 1, <1 time/week = 2, 1–3 times/week = 3, 4–6 times/week = 4, and once a day or more = 5. Responses were coded, summed, and averaged to create a score for each food type or activity.

Fathers were asked three questions about the frequency of practices regarding making fruit, vegetables, SSBs, fast food, sweets/salty snacks, and physical activity available at home. Availability of screen time opportunities was assessed with only one question. Making fruit and vegetables available at home was assessed separately by frequency of fathers (1) buying, (2) preparing, and (3) making sure adolescents had different kinds of fruits and vegetables. Making SSBs, sweets/salty snacks, and fast food available at home was assessed separately for each type of food/beverages by the frequency of fathers (1) buying, (2) preparing, and (3) giving money to adolescents to buy these foods. Making physical activity available was assessed by the frequency of fathers (1) taking their adolescent to a place he/she can be physically active, (2) sending their adolescent outside to be physically active when the weather is nice, and (3) making opportunities available for their adolescent to be physically active. Making screen time available was assessed by the frequency of fathers making screen time opportunities available to their adolescents. Response options for all availability questions were almost never or never = 1, rarely = 2, sometimes = 3, often = 4, and almost always or always = 5. The responses to the three questions for each lifestyle behavior, except for screen time, were coded, summed, and averaged to create an availability score.

### 2.7. Data Analysis

Initial sample size and power calculations were completed based on expected primary program outcomes as described elsewhere [29]. Post hoc power calculations were completed using nQuery sample size software (Version 4.0.0.0) to determine the power available to detect the observed between-group differences in parenting practice outcomes as significant at alpha = 0.05, using the study sample size in each group.

All fathers who had both Time-1 and Time-2 data were included in the analysis of parenting practice frequency. The first aim of the analysis was to describe observed changes in parenting practices. Descriptive statistics for Time-1 sociodemographic characteristics and Time-1-to-Time-2 changes for parenting practices for intervention and delayed-treatment control groups were assessed using independent two-sample *t*-tests, Chi-square and Fisher’s exact tests.

Linear regression mixed models were used to address the second aim, which was to assess adjusted differences in mean change from Time-1 to Time-2 in father parenting practices outcomes between the intervention and delayed-treatment control groups. The mixed models were adjusted for father age and adolescent sex and age. Additionally, the models included a random intercept for sites and a random intercept for fathers nested within sites to account for clustering of fathers within sites.

All analysis was performed using SAS software version 9.4 (Cary, NC, USA, 2002–2012) with statistical significance defined as *p* < 0.05.

## 3. Results

The retention rate was 64% for father/adolescent dyads based on withdrawal from the study because of relocation, scheduling conflicts, or loss to follow-up. Mean BMI for the fathers who completed Time-1 and Time-2 data collection (*n* = 94) was 29.7 vs. 28.3 for the fathers who only completed Time-1 data collection (*p* = 0.041). Adolescent sex (*p* = 0.013) was significantly different between those whose fathers completed both Time-1 and Time-2 data collections and those whose fathers only completed Time-1 data collection.

Of 147 father/adolescent dyads, 77 were randomized into the intervention group, and 70 were randomized into the delayed-treatment control group. Random assignment was not followed correctly by 11 father/adolescent dyads (4 father/adolescent dyads randomized to intervention attended the delayed-treatment control group educational sessions, while 7 father/adolescent dyads randomized to the delayed-treatment control group attended the intervention group). Therefore, there were 80 father/adolescent dyads in the intervention group educational sessions, and 67 father/adolescent dyads in the delayed-treatment control group educational sessions. Of the 94 fathers/adolescent dyads who completed both Time-1 and Time-2 data, 48 were in the intervention group, and 46 were in the delayed-treatment control group.

Overall, the demographic characteristics of fathers and adolescents in the intervention and delayed-treatment control groups were similar at Time-1 (Table 1). The mean father age was 42.1 (7.4) years. Most fathers reported having a yearly household income of ≤$49,999 (87%), completing high school or less (77%), being employed full-time (72%), speaking exclusively to primarily Spanish at home (81%), being married (86%), and having lived in the U.S. for more than 10 years (98%). The mean BMI of all the fathers was 29.7 kg/m^2^. The mean age of the adolescents was 12.2 (1.4) years, with 62% being male and 38% female. The mean BMI percentile of all adolescents was 77.6.

The means and standard deviations of Time-1 parenting practices were reported for the treatment groups in Table 2. The Time-1 intervention fathers’ frequency of fruit role modeling was significantly lower than that of the fathers in the delayed-treatment control group (*p* = 0.020). Also, the Time-1 frequency of screen time role modeling was significantly higher in the intervention fathers compared to the delayed-treatment control group fathers (*p* = 0.047). From Time-1 to Time-2, the intervention fathers reported an increased mean for fruit role modeling frequency (Mean = 0.44, SD = 0.97, *p* = 0.001) and a decreased mean for screen time modeling frequency (Mean = –0.22, SD = 1.18, *p* = 0.028) compared to the fathers in the delayed-treatment control group, based on unadjusted tests for paired data (Table 3).

After adjusting for covariates (Table 4), the intervention fathers had a significantly increased adjusted mean for fruit modeling frequency and decreased adjusted mean for screen time modeling frequency compared to the delayed-treatment fathers [group*time (SE) = 0.63 (0.19), *p* = 0.002 for fruit modeling; group*time (SE) = −0.49 (0.24), *p* = 0.039 for screen time modeling] based on linear regression mixed models (Table 4).

## 4. Discussion

This randomized controlled trial examined the effects of the Padres program on Latino fathers’ food and activity parenting practices. Compared to the delayed treatment control group fathers, the intervention group fathers reported a higher adjusted mean change for fruit modeling frequency and a lower adjusted mean change for screen time modeling frequency from Time-1 to Time-2. Overall, this study identified a moderate positive intervention effect for 2 of 14 parenting practices (adjusted models), including fruit and screen time role modeling. In other studies, role modeling behaviors were associated with greater healthy food consumption [16] and with sedentary activity [40,41] among children and adolescents.

A limited number of studies have examined improvements in Latino food parenting practices in community-based programs, resulting in few studies available for comparison to the current study. One study with primarily low-income Hispanic parents (95% mothers) and children (3–11 years) showed Time-1 to Time-2 intervention improvements in two parenting practices involving food availability [42] but not in frequency of food intake role modeling. This finding was in contrast to the current study, where the intervention group fathers had a mean increase in fruit modeling frequency and a mean decrease in screen time modeling frequency compared to the delayed-treatment control group fathers. The previous intervention was a pilot study [42] with primarily mothers and did not include a control group; therefore, the results were not directly comparable to the results of the current study.

The current study demonstrated no intervention effects for most of the parenting practices, which could be related to ceiling effects. The majority of the fathers in both groups reported a high frequency of healthful food and activity practices and a low frequency of most of the unhealthful food and activity practices at Time-1, except for screen time modeling and availability. For example, intervention fathers reported role modeling screen time more than 1–3 times a week and role modeling sweets/salty snacks and fast food intake less than once a week. Thus, fathers who reported a high frequency of unhealthful food and activity practices before the intervention may have been better able to apply what they learned during the intervention to improve the frequency of some parenting practices.

Another possible explanation for not observing improvements for most parenting practices in the current study could be associated with social determinants of health. Evidence from previous studies demonstrates that being part of a low-income household and having lower educational attainment are two key social factors associated with poor health in the United States [43]. Racial and ethnic minorities with low socioeconomic status, including the Hispanic/Latino population, often experience health disparities [43,44]. The majority of fathers in this study had a high school diploma or less (79%) and had lower-incomes (87%), even though most had full-time employment. The role of fathers as family providers with busy work schedules may have kept fathers from implementing parenting practices. For example, limited resources to purchase healthy foods may have restricted the ability to make healthy foods available at home and/or role model healthy food intake. Also, fathers who work long hours may have limited time to interact with adolescents and apply parenting practices; thus, a longer period of time from post-intervention to the completion of evaluation surveys might have allowed fathers to better apply the practices promoted in the program.

This study had several limitations. The COVID-19 pandemic did not allow for continued in-person program implementation after March 2020, thus limiting the sample size. The low retention rate and smaller sample size than expected resulted in the study being underpowered to detect significant changes in most of the parenting practice comparisons. The Padres program was only implemented in community centers and churches in the Minneapolis/St. Paul metropolitan area and only with low-income families, which limited the generalizability of study findings to the broader Latino/Hispanic population. Defining groups by randomization (not by group assignments) might cause bias in the group comparisons, since the randomization assignments were not followed correctly for eleven dyads. Not correctly following assignments may have occurred because participants in some locations may have known each other and preferred to attend sessions with relatives or friends or needed to share transportation. However, similar results were obtained from a sensitivity analysis when the group comparisons were defined by participation instead of randomization. Another potential limitation is that delayed-treatment control participants may have been exposed to the intervention, since participants may have known each other. Also, participants may have enrolled in this study because of an interest in nutrition and health and/or financial compensation, which could have biased the intervention data.

## 5. Conclusions

The current study showed positive adjusted Time-1 to Time-2 change in two parenting practices between groups, including an adjusted mean increase in fruit role modeling frequency and an adjusted mean decrease in screen time modeling frequency among intervention group participants compared to delayed-treatment control group participants. Overall, only 2 of 14 Latino father parenting practices were reported to be improved after the intervention. The lack of significant findings for other parenting practices may be associated with the limited sample size, low family socioeconomic status, and possible ceiling effects of baseline paternal parenting practices. Future studies could consider social determinants of health and family strengths when developing interventions to support an increase in healthy Latino father parenting practices.

## Figures and Tables

**Table 1:** Time-1 father and adolescent demographic characteristics (*n* = 94).

Demographic Characteristics	All *n* = 94	Intervention *n* = 48	Delayed Treatment Control *n* = 46	*p*-Values
**Father demographics**				
Age, mean (SD ^1^)	42.1 (7.4)	43.1 (7.1)	41.1 (7.7)	0.195 ^2^
Annual income, *n* (%)				
<$25,000	38 (41.8)	23 (48.9)	15 (34.1)	0.229 ^3^
$25,000–<$50,000	41 (45.0)	20 (42.6)	21 (47.7)	
≥$50,000	12 (13.2)	4 (8.5)	8 (18.2)	
Marital status, *n* (%)				
Married	78 (85.7)	41 (87.2)	37 (84.1)	0.674 ^4^
Living with partner	6 (6.6)	2 (4.3)	4 (9.1)	
Single/widowed/divorced/separated	7 (7.7)	4 (8.5)	33 (6.8)	
Education, *n* (%)				
Middle school or less	33 (35.9)	20 (41.7)	13 (29.6)	0.410 ^3^
HS ^1^ grad or GED ^1^	38 (41.3)	17 (35.4)	21 (47.7)	
College (any) or technical school	21 (22.8)	11 (22.9)	10 (22.7)	
Employment, *n* (%)				
Self-employed	10 (11.1)	5 (10.6)	5 (11.6)	0.643 ^4^
Unemployed/homemaker	5 (5.6)	3 (6.4)	2 (4.7)	
Part-time employment	8 (8.9)	6 (12.8)	2 (4.7)	
Full-time employment	67 (74.4)	33 (70.2)	34 (79.1)	
Years in the US, *n* (%)				
<10	2 (2.2)	1 (2.1)	1 (2.3)	0.834 ^4^
10–<20	52 (57.1)	29 (61.7)	23 (52.3)	
20–<30	33 (36.3)	15 (31.9)	18 (40.9)	
≥30	4 (4.4)	2 (4.3)	2 (4.6)	
Language, *n* (%)				
More Spanish than English	76 (81.7)	37 (77.1)	39 (86.7)	0.515 ^4^
Equal Spanish and English	15 (16.1)	10 (20.8)	5 (11.1)	
More English than Spanish	2 (2.2)	1 (2.1)	1 (2.2)	
Father BMI ^1^ (kg/m^2^), mean (SD ^1^)	29.7 (3.7)	29.5 (4.0)	29.8 (3.4)	0.728 ^2^
**Adolescent demographics**				
Age, mean (SD ^1^)	12.2 (1.4)	12.2 (1.5)	12.1 (1.3)	0.778 ^2^
Sex, *n* (%)				
Male	58 (61.7)	33 (68.8)	25 (54.3)	0.151 ^3^
Female	36 (38.3)	15 (31.2)	21 (45.7)	
BMI ^1^ percentile ^5^, mean (SD ^1^)	77.6 (23.8)	80.3 (21.2)	74.7 (26.2)	0.260 ^2^

^1^ SD = Standard Deviation, HS = High School, GED = General Educational Development test, BMI = body mass index; ^2^ Two sample *t*-test; ^3^ Chi-square test; ^4^ Fisher’s exact test; ^5^ Adolescent BMI percentiles for age and sex were calculated from SAS codes based on the 2000 CDC Growth Charts; *p*-value < 0.05.

**Table 2:** Father-reported paternal parenting practices: Time-1 means and standard deviations for the intervention and delayed-treatment control groups (*n* = 94).

Paternal Parenting Practices	N ^1^	All *n* = 94	Intervention *n* = 48	Control *n* = 46	*p*-Values
**Role modeling ^3^ times/week, mean (SD ^2^)**					
Fruit intake	88	3.19 (0.93)	2.95 (0.97)	3.41 (0.97)	0.020 *
Vegetable intake	91	3.21 (0.98)	3.15 (0.98)	3.27 (0.99)	0.549
SSBs ^2^ intake	94	2.32 (1.03)	2.36 (1.04)	2.27 (1.02)	0.663
Sweets/salty snack intake	94	1.87 (0.86)	1.89 (0.89)	1.86 (0.84)	0.882
Fast food intake	92	1.85 (0.70)	1.75 (0.71)	1.95 (0.69)	0.183
Physical activity	90	2.75 (1.13)	2.82 (1.19)	2.67 (1.06)	0.516
Screen time	93	2.82 (1.10)	3.04 (1.14)	2.59 (1.02)	0.047 *
**Make available ^4^, mean (SD ^2^)**					
Fruit	94	4.06 (0.74)	4.09 (0.63)	4.03 (0.83)	0.672
Vegetables	93	3.96 (0.84)	4.03 (0.82)	3.88 (0.86)	0.370
SSBs ^2^	93	1.77 (0.60)	1.80 (0.60)	1.75 (0.60)	0.660
Sweets/salty snacks	94	1.81 (0.66)	1.88 (0.63)	1.74 (0.68)	0.306
Fast food	94	1.90 (0.66)	1.93 (0.71)	1.86 (0.61)	0.323
Physical activity	93	3.77 (0.93)	3.78 (0.86)	3.77 (1.00)	0.970
Screen time	92	3.10 (1.09)	3.23 (1.15)	2.96 (1.02)	0.223

^1^ N reported for each outcome; ^2^ SSB = Sugar-sweetened beverages, SD = Standard Deviation; ^3^ Role modeling frequency for fruit, vegetable, SSB, fast food, and sweets/salty snack consumption and physical activity and screen time was based on the average of two items with response options: almost never or never, <1 time/week, 1–3 times/week, 4–6 times/week, and once a day or more; ^4^ Frequency of making fruit, vegetable, SSB, fast food, and sweets/salty snack consumption and physical activity and screen time available at home was based on the average of three items with response options: almost never or never = 1, not often = 2, sometimes = 3, often = 4, and almost always or always = 5. * Indicates significant differences between groups. *p*-value < 0.05.

**Table 3:** Time-1 to Time-2 changes in paternal food and activity parenting practices outcomes.

Paternal Parenting Practices ^1^	N ^2^	All *n* = 94	Intervention *n* = 48	Control *n* = 46	*p* Values
**Role modeling times/week, mean (SD ^3^)**					
Fruit intake	82	0.12 (0.95)	0.44 (0.97)	−0.23 (0.80)	0.001 *
Vegetable intake	88	0.10 (1.07)	0.20 (0.98)	0.00 (1.17)	0.396
SSBs ^3^ intake	91	−0.36 (1.02)	−0.45 (1.07)	−0.27 (0.98)	0.407
Sweets/salty snack intake	94	−0.09 (0.85)	−0.19 (0.87)	0.01 (0.81)	0.258
Fast food intake	90	−0.12 (0.65)	−0.16 (0.65)	−0.08 (0.67)	0.575
Physical activity	90	0.01 (0.97)	0.14 (0.97)	−0.14 (0.96)	0.175
Screen time	91	0.04 (1.21)	−0.22 (1.18)	0.33 (1.18)	0.028 *
**Make available, mean (SD ^3^)**					
Fruit	93	−0.00 (0.68)	0.06 (0.69)	−0.06 (0.68)	0.405
Vegetables	93	0.02 (0.84)	0.05 (0.88)	−0.00 (0.81)	0.766
SSBs ^3^	93	−0.04 (0.78)	−0.05 (0.88)	−0.04 (0.81)	0.934
Sweets/salty snacks	94	−0.12 (0.99)	−0.25 (0.90)	0.01 (1.07)	0.204
Fast food	94	0.10 (0.81)	−0.03 (0.82)	0.24 (0.79)	0.108
Physical activity	92	0.02 (0.78)	0.02 (0.79)	0.02 (0.79)	0.991
Screen time	92	−0.05 (1.22)	−0.04 (1.37)	−0.07 (1.05)	0.925

^1^ Two-sample *t*-test of difference in means; ^2^ N reported for each outcome; ^3^ SSB = Sugar-sweetened beverages, SD = Standard Deviation; * Indicates significant differences between groups. *p*-value < 0.05.

**Table 4:** Adjusted group differences for Time-1 to Time-2 change in paternal food and activity parenting practice outcomes.

Positive ^2^ Paternal Parenting Practices (Time-1 to Time-2 Change)	Estimate (SE ^1^) and *p*-Value for Fixed Effects from Mixed Model ^2^ with Random Intercept for Site and Random Intercept for Father Nested Within Site			
	Group ^3^ (Ref = Control)	Time ^4^ (Ref = Time-1)	Group * Time ^5^ (Ref = Control Time-1 to Time-2 Change)	*p* Values for Group * Time
**Role modeling**				
Fruit intake	−0.31 (0.16)	−0.18 (0.14)	0.63 (0.19)	0.002 *
Vegetable intake	−0.12 (0.17)	0.05 (0.15)	0.18 (0.21)	0.414
SSBs ^1^ intake	−0.03 (0.17)	−0.33 (0.15)	−0.11 (0.21)	0.598
Sweets/salty snack intake	0.01 (0.14)	0.00 (0.12)	−0.17 (0.17)	0.298
Fast food intake	−0.07 (0.12)	−0.09 (0.09)	−0.11 (0.13)	0.395
Physical activity	0.04 (0.18)	−0.11 (0.14)	0.29 (0.20)	0.142
Screen time	0.38 (0.19)	0.28 (0.17)	−0.49 (0.24)	0.039 *
**Make Available**				
Fruit	−0.07 (0.14)	−0.05 (0.10)	0.16 (0.14)	0.254
Vegetables	0.01 (0.15)	0.02 (0.12)	0.10 (0.17)	0.566
SSBs ^1^	−0.04 (0.12)	−0.11 (0.11)	0.03 (0.16)	0.854
Sweets/salty snacks	0.11 (0.12)	0.01 (0.13)	−0.25 (0.18)	0.174
Fast food	0.02 (0.12)	0.19 (0.11)	−0.22 (0.16)	0.160
Physical activity	−0.21 (0.15)	0.01 (0.11)	0.07 (0.16)	0.672
Screen time	0.13 (0.17)	−0.16 (0.17)	0.09 (0.24)	0.700

^1^ Abbreviations: SE = standard error, SSB = sugar-sweetened beverages; ^2^ Models were adjusted for father age, adolescent age and sex; ^3^ Group effect estimates the adjusted difference between intervention and control means across both times; ^4^ Time effect estimates the adjusted difference between Time-1 and Time-2 means across both groups; ^5^ Group*time estimates the adjusted difference in mean change from Time-1 to Time-2 for intervention compared to control; * Indicates significant differences in adjusted Time-1 to Time-2 changes between intervention and control at *p* < 0.05.

## Data Availability

The de-identified data and SAS codes used in this study are available on request from the corresponding author. The data are not publicly available because the data analysis phase of the study is still currently being completed, while the intervention and all data collection have been completed.
